# Understanding worldwide skin atopy across regions. Environmental, cultural, genetic, and lifestyle factors

**DOI:** 10.3389/fmed.2026.1796337

**Published:** 2026-04-01

**Authors:** Caroline Jacobzone-Lévêque, Rieko Tsubouchi, Francisco Salazar, Haiping Zhang, Dimitre Dimitrov

**Affiliations:** 1 Department of Dermatology, Hôpital du Scorff, Lorient, France; 2 Ginza Skin Clinic, Tokyo, Japan; 3 Department of Dermatology, Nippon Medical School, Tokyo, Japan; 4 Department of Pediatric Dermatology, Instituto Dermatológico de Jalisco, Guadalajara, Mexico; 5 Department of Dermatology, Xuanwu Hospital, Capital Medical University, Beijing, China; 6 Department of Dermatology, Sheikh Khalifa Medical City, Abu Dhabi, United Arab Emirates; 7 Khalifa University, Abu Dhabi, United Arab Emirates

**Keywords:** atopic skin, environmental, lifestyle, skin barriers, vitamin D

## Abstract

Cutaneous atopy, which predominantly manifests as atopic dermatitis (AD), represents a significant global health concern due to its high prevalence and profound impact on patients’ quality of life. AD affects up to 20% of children and 10% of adults worldwide, with a rising incidence in both industrialized and developing regions. While genetic predisposition is a key determinant, most existing literature analyzes risk factors in isolation, limiting a comprehensive understanding of the disease. This article provides an integrative, regionally informed perspective on how environmental, cultural, genetic, and lifestyle factors may interact to shape the clinical expression of cutaneous atopy in diverse populations and regions. Drawing on published evidence and insights from an international expert panel, we highlight the complexity and multifactorial nature of the atopic environment, emphasizing the simultaneous disruption of the four key skin barriers—physical, chemical, immunological, and microbial—in AD. The article further addresses how regional differences in barrier function and the influence of urban versus rural living conditions can affect disease manifestation, underlining the need for personalized and holistic therapeutic strategies. This integrative perspective aims to offer healthcare professionals an updated framework for the management of AD, enabling more effective interventions tailored to the realities of diverse patient populations.

## 1 Introduction

Skin atopy refers to a genetic predisposition to develop atopic conditions, particularly atopic dermatitis (AD), and is characterized by a dysregulated immune response and impaired skin barrier. This skin alteration represents a growing public health concern worldwide. In developed countries, the prevalence of atopy ranges from 10% to 30% (1). Twin and epidemiologic studies, together with family and animal experiments, indicate that genetics is one of the major determinants in the development of atopy (1). AD is one of its earliest and most prevalent clinical manifestations, affecting up to 20% of children and 10% of adults globally, with increasing incidence across both industrialized and developing nations (2). This chronic, relapsing inflammatory skin disease is characterized by intense pruritus, barrier dysfunction, and immune dysregulation, significantly impacting patients’ quality of life and placing a considerable burden on healthcare systems (3).

Although the literature on the prevalence and key determinants of atopic skin is extensive, most studies have examined risk factors in isolation. Here, we provide a consolidated, regionally informed narrative synthesis that integrates published evidence with expert interpretation from different geographic settings to illustrate how environmental, cultural, genetic, and lifestyle factors may interact across populations. This approach aims to connect established concepts with more recently recognized influences and to highlight the complexity and multifactorial nature of the “atopic environment,” and its impact on disease expression. A brief description of the methodological approach is provided below, and additional details are available in the Supplementary material. This integrated perspective, presented here as a regionally informed synthesis, may offer healthcare professionals a framework to adopt more targeted and holistic strategies tailored to the realities of diverse patient populations.

**FIGURE 1:**
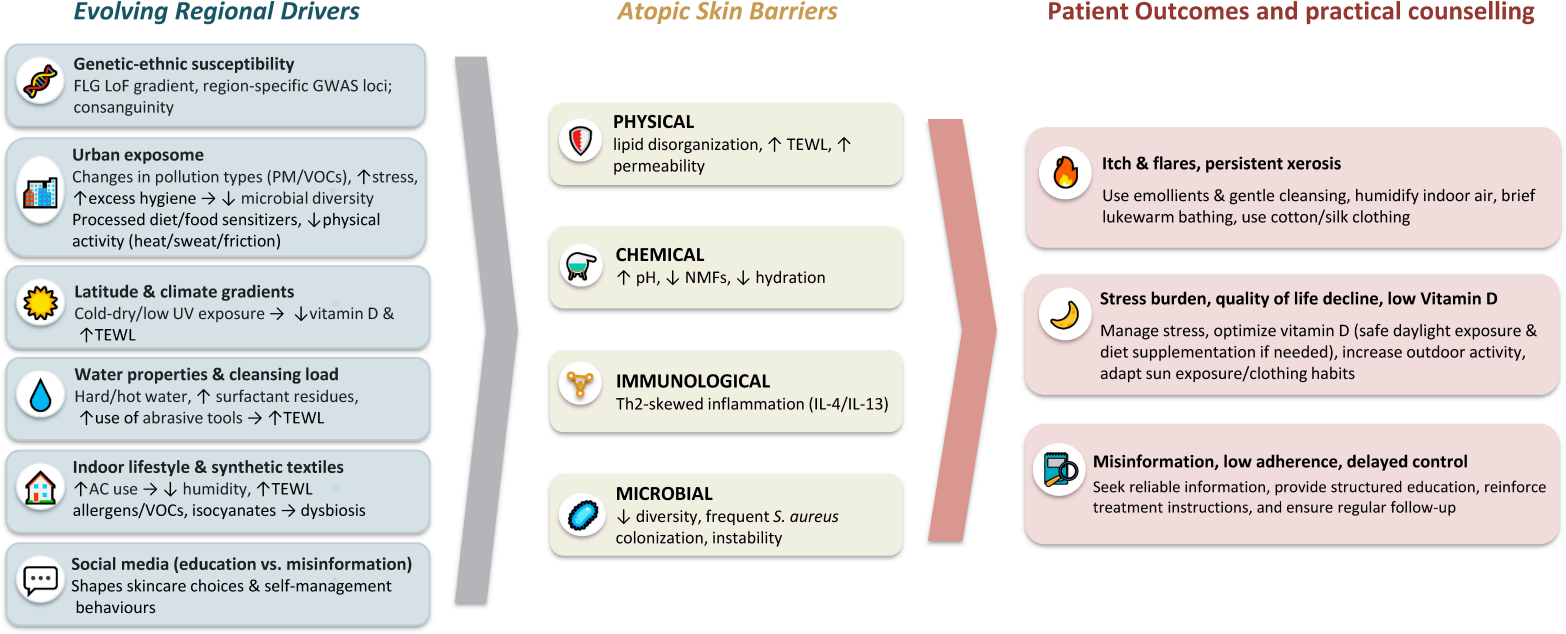
Evolving regional drivers shaping atopic dermatitis: barrier-specific effects and patient-centered practical counseling. AC, air conditioning; AD, atopic dermatitis; FLG, filaggrin; GWAS, genome-wide association studies; IL-13, interleukin 13; IL-4, interleukin 4; LoF, loss-of-function; NMFs, natural moisturizing factors; PM, particulate matter; QoL, quality of life; *S. aureus*, *Staphylococcus aureus*; TEWL, transepidermal water loss; Th2, T helper 2; UV, ultraviolet; VOCs, volatile organic compounds.

An overview of the evolving factors, the mechanistic pathways affecting the four skin barriers, and related counseling implications is summarized in Figure 1.

## 2 Methodological approach

This manuscript was developed as a narrative, regionally informed review intended to raise awareness of how environmental, cultural, genetic, and lifestyle factors may shape atopic skin across diverse regions. A pragmatic, targeted search was conducted in PubMed, Scopus, and Google Scholar with no start date restriction; the last search was conducted in November 2025. Search strings combined core terms for atopic skin/AD and the skin barrier with region relevant exposures (e.g., climate/latitude, urbanization/air pollution, water hardness and bathing practices, textiles, vitamin D, psychosocial stress, and skin microbiome). Evidence was selected for relevance to the manuscript’s aims and included narrative and systematic reviews, observational and population-based studies, and clinical studies or meta-analyses when directly informative.

The five authors of the manuscript constituted the expert panel. Before a dedicated online meeting held on 17 March 2025, the authors reviewed the literature within their assigned thematic areas and completed a brief pre-meeting questionnaire containing topic-specific guiding questions. These prompts were used to structure a focus-group-style discussion during which region-specific observations, clinical perspectives, and literature-based evidence were examined within the four-barrier framework. This process was intended to support interpretive synthesis rather than to generate formal consensus statements and did not follow a Delphi methodology or predefined voting thresholds. Differences in interpretation were addressed through discussion during the meeting, and minor wording or framing issues arising during manuscript development were resolved subsequently by e-mail. The guiding questions are provided in Supplementary Table 1.

## 3 Why environmental and regional factors may have an impact on atopic skin: the four skin barriers

It is well recognized that various factors, including environmental ones, can significantly impact skin barrier function. This raises the following question: Should we anticipate regional differences in skin barrier impairment, particularly in atopic skin, given the diverse environmental and cultural conditions worldwide?

The cutaneous barrier is a multilayered defense system comprising physical, chemical, immunological, and microbial domains that act together to protect against external insults (4). Their efficiency can vary regionally, influenced by environment, cultural practices, and genetic–ethnic factors. In atopic skin, these domains show characteristic alterations: the physical barrier (stratum corneum) exhibits lipid disorganization and increased transepidermal water loss (TEWL) (5–7); the chemical barrier (acid mantle and natural moisturizing factors [NMFs]) shifts toward higher pH and reduced hydration (8); the immunological barrier is skewed to a T helper 2 (Th2) profile with cytokines such as interleukin 4 (IL4) and interleukin 13 (IL13) (9); and the microbial barrier loses diversity, often with *Staphylococcus aureus* (*S. aureus*) colonization (10). Table 1 summarizes the core components, typical readouts, and hallmark alterations for each domain.

**TABLE 1:** The skin barrier as a multilayered system and hallmark alterations in atopic skin (4).

Barrier domain	Core structural/functional components	Typical readouts (metrics)	Hallmark alterations in atopic skin	References
Physical	Stratum corneum corneocytes; intercellular lipids (ceramides, cholesterol, FFAs); corneodesmosomes	TEWL; lipid profile; corneocyte cohesion	↑ TEWL; lipid disorganization; increased permeability	Palmer et al. (5); Basu et al. (6); Rosso et al. (7)
Chemical	Acid mantle (surface pH); NMFs; buffer capacity	Surface pH; NMFs (e.g., PCA, UCA); buffering curves	↑ pH (less acidic); ↓ NMFs; impaired hydration	Baker et al. (8)
Immunological	Innate/adaptive immune interface; cytokine milieu; neuroimmune crosstalk	Cytokines (IL4, IL13); Th2 signature; immune peptides	Th2 dominant skew; inflammatory amplification	Suárez-Fariñas et al. (9)
Microbial	Community composition/diversity; commensal–pathogen balance	α/βdiversity indices; *S. aureus* carriage	↓ diversity; frequent *S. aureus* colonization; instability	Koh et al. (10)

↑ Increased and ↓ decreased. FFAs, free fatty acids; IL-4, interleukin-4; IL-13, interleukin-13; NMFs, natural moisturizing factors; PCA, pyrrolidone carboxylic acid; *S. aureus*, *Staphylococcus aureus*; TEWL, transepidermal water loss; Th2, T helper 2 cells; UCA, urocanic acid.

Several regionally patterned exposures discussed in this review can plausibly influence one or more barrier domains. For example, urban air pollution may promote oxidative stress and inflammatory signaling, with downstream effects on the immunological barrier and microbial composition (11); psychosocial stress can activate neuro-immune pathways and impair barrier repair (12); and vitamin D influences epidermal differentiation and antimicrobial peptide production, impacting the immunological, physical, and microbial skin barriers (13, 14). These mechanistic links are summarized, together with practical counseling implications, in Figure 1.

## 4 Regional intrinsic factors impacting atopic skin

### 4.1 Genetic component: filaggrin mutations across regions

Filaggrin (FLG), encoded in the epidermal differentiation complex on chromosome 1q21, is a key structural protein expressed in the stratum corneum, where it aggregates keratin filaments and contributes to the formation of a compact, mechanically resilient barrier. Its degradation products form part of the NMFs, which help maintain hydration and an acidic pH. Loss-of-function (LoF) variants in the FLG gene impair these processes, leading to increased TEWL, elevated surface pH, and heightened susceptibility to irritants and allergens. These alterations position FLG as a major genetic determinant of skin barrier dysfunction in atopic skin. Building on this functional role, multiple studies have examined the prevalence of FLG LoF mutations across regions and their contribution to AD susceptibility (Table 2). A systematic review estimated global prevalence at 19.1% in AD patients versus 5.8% in controls (15), showing a clear latitudinal gradient, with higher prevalence in northern areas and minimal presence in tropical populations (16). Europe showed the highest prevalence, Asia a moderate prevalence, and the Middle East and Africa the lowest prevalence (Table 2).

**TABLE 2:** Prevalence of the filaggrin loss-of-function mutation across regions.

Region/population	FLG LoF prevalence in AD patients	Notes/context	References
Global (systematic review)	19.1% (AD) vs. 5.8% (controls)	Confirms strong association but not universal	Khatib et al. (15)
Europe (severe AD)	Up to 50%	Highest reported prevalence	Criado et al. (91)
East Asia–Korea	15.7%	Moderate prevalence	On et al. (92)
East Asia–Japan	27%	Higher than Korea	Teye et al. (93)
Middle East–Iran	Rare/absent	Complete sequencing found no common LoF	Hassani et al. (94)
Africa–Ethiopia	Absent (common LoF); 1 novel variant (632del2)	Only 1 case in 103 patients	Winge et al. (95)

AD, atopic dermatitis; LoF, loss-of-function mutation.

Although FLG mutations increase susceptibility, they are neither necessary nor sufficient for disease development (17). Genome-wide association studies (GWAS) have identified additional loci on chromosomes 11q13, 19p13.2, and 5q31.1, as well as Asianspecific variants at 5q22.1 and 20q13.33 (18, 19). These findings underscore the ethnic heterogeneity and support a multifactorial etiology involving barrier dysfunction, immune regulation, and vitamin D metabolism (20, 21) (Table 3). In this context, evidence suggested a functional interaction between FLG biology and vitamin D pathways (16). FLG breakdown products influence the cutaneous ultraviolet (UV) response needed for endogenous vitamin D synthesis, while vitamin D itself modulates barrier immunity through the regulation of antimicrobial peptides and Th2/Treg balance. This link reinforces the idea that intrinsic genetic factors such as FLG status may interact with regionally patterned environmental exposures to shape the immunological phenotype of atopic skin.

**TABLE 3:** Loci associated with atopic dermatitis identified through genome-wide association studies: functional grouping and barrier domains.

Chromosomal locus	Key genes	Primary biological function	Barrier domain most impacted	References
11q13/11q13.1	*C11orf30*, *LRRC32*, *OVOL1*	Epithelial differentiation, transcriptional control	Physical (stratum corneum integrity)	Paternoster et al. (18); Esparza-Gordillo et al. (96)
19p13.2	*ACTL9*, *ADAMTS10*	Extracellular matrix organization	Physical/chemical	Paternoster et al. (97)
5q31.1	*IL4*, *IL13*, *RAD50*, *KIF3A*	Inflammatory and immune regulation, Th2 skew, apoptosis	Immunological	Esparza-Gordillo et al. (96)
5q22.1	*TMEM232*, *SLC25A46*	Membrane transport, signaling	Chemical/Immunological	Sun et al. (19)
20q13.33	*TNFRSF6B*, *ZGPAT*	Apoptosis modulation, immune signaling	Immunological	Sun et al. (19)
Vitamin D pathway genes	Various	Hormone metabolism, receptor activity	Chemical/Immunological	Nedoszytko et al. (29)

### 4.2 Consanguinity: cultures that promote intrafamilial marriage

Consanguinity may contribute to a higher genetic predisposition to AD by increasing homozygosity and the concentration of atopy-related genetic variants within families, although current evidence has not yet established a definitive causal relationship. Notably, studies conducted in Saudi Arabia, where most of the existing literature originates, suggest that the high prevalence of consanguineous marriages in the region may be a contributing factor to the increased incidence of AD among children (22–24).

## 5 Regional extrinsic factors impacting atopic skin

### 5.1 Urban versus rural lifestyles

A growing body of evidence suggests that urban living, which is continuing to rise, is associated with an increased risk of skin atopy, particularly in children. A review of 43 studies and 1.7 million participants found a significantly higher risk of AD in urban children (odds ratio [OR] = 1.55), while adults showed a similar but non-significant association (OR = 1.29) (25). This disparity is consistent with findings from China (26, 27), with stronger effects in developing (OR = 1.95) compared to developed countries (OR = 1.35) (25). A Polish study noted a higher prevalence of AD in urban adults, but not in children (28).

Environmental and lifestyle factors—air pollutants, hygiene, reduced microbial exposure, and stress—may explain this trend. Pollutants like particulate matter (PM), volatile organic compounds (VOCs), traffic-related pollution (29), tobacco smoke, and heavy metals disrupt the skin barrier and trigger immune responses (11). Conversely, some rural microbial exposures, including endotoxin-rich environments, may confer a degree of immune protection in certain contexts (30). Urban living is also associated with sedentary behavior and reduced physical activity (31, 32), though some debate exists (33). Prolonged indoor time may reduce sunlight exposure and vitamin D synthesis, which is important in the pathophysiology of AD. Psychological stress, prevalent in urban settings, is a known AD trigger. Chronic stress activates immunological and neuroendocrine pathways, compromising the skin barrier and promoting Th2 inflammation (12). AD patients report more stress and poorer emotional quality of life (34), and maternal stress is linked to higher AD risk in offspring (35).

Urban environments also show reduced skin microbiome diversity, weakening the microbial barrier (36–38). These disruptions reflect broader ecological models of reduced microbial contact and increased pollutant exposure. Excessive hygiene in urban areas further disrupts the microbiota and barrier function, and skews immune responses toward a Th2 profile, increasing the risk of atopic disease (39).

In addition, dietary patterns, levels of physical activity, and exposure to food allergens may differ between urban and rural environments. Urbanization is often associated with more processed diets, reduced physical activity, and greater diversity of potential food sensitizers, whereas rural settings may present different nutritional habits and allergen profiles. For example, in Japan, particularly in urban settings, ready-to-eat meals from convenience stores and supermarkets are highly accessible and frequently consumed, raising clinical concern about greater exposure to processed foods and food additives as potential lifestyle-related factors relevant to AD, although direct evidence remains limited. In addition, published studies suggest that dietary patterns may influence AD through metabolic inflammation, the gut–skin axis, and microbiome-linked immune modulation (40), while some of the regional observations presented are derived from the clinical experience of the contributing experts. Patients with AD have higher rates of food allergies; however, routine broad testing or empiric elimination diets should not be initiated without a compatible clinical history, as unnecessary restrictions might impair nutrition without improving skin outcomes (41). Although physical activity is generally beneficial, heat, sweat, and friction may exacerbate symptoms. In this context, practical advice includes breathable clothing, prompt showering with gentle cleansers, and immediate emollient application (42).

In summary, these urban-rural differences in stress, pollution, microbial imbalance, diet, food-allergen exposure, physical activity and hygiene practices may collectively impair immune regulation and skin barrier function, contributing to the burden of atopic skin conditions in urban settings.

### 5.2 Water properties and hygiene traditions across cultures worldwide

#### 5.2.1 The role of water properties

In several European regions, water properties, particularly water hardness and temperature, have been identified as potentially relevant environmental factors influencing skin atopy (43).

When assessing hard water, calcium carbonate is the principal mineral indicator of hardness, usually containing more than 200–250 mg/L of this component. However, this defining criterion may vary depending on the geographic region (Table 4). Elevated levels of calcium carbonate in water have been shown to impact skin barrier function. It is generally understood that increasing water temperature improves the solubility of calcium carbonate, which would theoretically reduce crystal formation and consequently minimize skin irritation.

**TABLE 4:** Typical municipal tap water hardness and category (mg/L as CaCO_3_).

Country	Representative data point (scope)	Hardness (mg/L as CaCO_3_)	Category	Source
China	National mean (314 cities; Ca & Mg converted to CaCO_3_)	≈152	Hard	National survey of Ca/Mg in public drinking water (we computed total hardness as CaCO_3_ using standard factors) (98)
France	Paris municipal supply (range across distribution networks)	≈230–280	Hard–very hard	ARS/Eau de Paris network, results reported in°f (≈23–28°f); 1°f ≈ 10 mg/L CaCO_3_ (99)
Japan	National mean (665 taps)/updated nationwide survey (2019–2024)	≈49–51	Soft	Nationwide hardness surveys (scientific reports, nature) (100, 101)
Mexico	Monterrey (official utility report; example of a large Mexican city)	≈251 (mean; 2014 S2)	Very hard	Monterrey water and drainage services, semi-annual report (total hardness as CaCO_3_) (102)
United Arab Emirates	Desalinated and remineralized (Dubai/Abu Dhabi; typical)	Typical range for remineralized desalinated water: 200–500 mg/L.	Hard	Utilities report desalinated water remineralization; numeric average not published (103)
United Kingdom	England and Wales	Varied, with large areas of ≈240–260 (zone means)	Hard–very hard	Reports with hardness (total) as CaCO_3_; wide regional variability (104)
United States	National context (USGS)	Varies by region; commonly 60–180+	Moderately hard–very hard	USGS hardness categories and national map; no single average recommended due to substantial regional variability (105)

Definition of water hardness and categories: Total hardness reported as mg/L CaCO_3_ and water classified as soft (0–60), moderately hard (61–120), hard (121–180), or very hard (> 180 mg/L), following USGS. For China, total hardness was computed from national mean calcium and magnesium concentrations using standard conversion factors (Hardness mg/L as CaCO_3_ = 2.5 × [Ca] + 4.1 × [Mg]). City/utility data points are used where national means are not reliable due to substantial intra-country variability.

However, when water is heated, the crystallization of other dissolved minerals is enhanced, which may lead to increased skin irritation upon contact. Furthermore, hard water increases the deposition of detergents such as sodium lauryl sulfate on the skin and promotes the formation of insoluble mineral-soap complexes. These residues are difficult to remove, compromise the skin barrier, and enhance irritant potential compared to soft water (44–46). When combined, these factors contribute to elevated TEWL, particularly when both water hardness and temperature are high.

Epidemiological studies suggest that while the prevalence of AD is higher in areas with hard water, there is no clear evidence of causation, suggesting that it may exacerbate rather than cause the condition (43), (47). However, in genetically predisposed individuals, particularly children with FLG gene mutations, which impair skin barrier integrity, exposure to hard water appears to further elevate the risk of AD (48). Despite these associations, randomized controlled trials have found no objective improvement in AD severity with the use of domestic water softeners (49). Thus, hard water should not be considered an isolated cause or a significant risk factor across all populations.

#### 5.2.2 Hot bathing across cultures

Hot bathing cultures, such as Japanese *onsen*, Korean *jjimjilbang*, Middle Eastern *hammams*, and thermal spas across Europe and Latin America, demonstrate a long-standing belief in the therapeutic value of heat, water, and ritual for overall wellbeing. From a dermatological perspective, immersion in warm, mineral-rich waters, when properly managed, may offer clinical benefits for individuals with atopic skin. These include the removal of allergens and pollutants, improved skin hydration, reduced inflammation, and modulation of the cutaneous microbiome through mineral exposure, particularly in waters rich in sulfur, magnesium, or bicarbonate (50–52) (Table 5). Additionally, psychological stress may be alleviated through the relaxing and communal aspects of these bathing rituals (53).

**TABLE 5:** Hot bathing traditions: water properties, protocols, dermatologic effects, and practical guidance for atopic skin.

Practice/region	Typical water properties and format	Typical temperature and exposure	Reported clinical effects in AD	Putative mechanisms (barrier/microbiome/ immune)	References
Japanese *onsen* (selected springs)	Often mineral-rich (e.g., sulfur-containing springs; some baths are acidic; others bicarbonate/mildly alkaline); immersion bathing	Frequently hot (> 42°C); exposure is often short but culturally frequent	Improvements reported in some cases; responses vary by spring type; benefit seen anecdotally/observationally; one report in an acidic hot spring for refractory AD	Mineral effects (e.g., sulfur) may reduce *S. aureus*; heat + minerals may modulate itch neuro-immune axis; pH and minerals may influence acid mantle and Langerhans cell activity	Proksch et al. (50); Matz et al. (51); Schempp et al. (52); Yosipovitch et al. (53)
Korean *jjimjilbang*/heat bathing	Heat rooms/saunas; variable mineral exposure; often dry or mixed humidity	High temp exposures interspersed with cooling; durations vary	Potential relief of pruritus/stress for some; risk of barrier dehydration if overdone	Heat may increase TEWL; stress reduction can help itch; no mineral-specific mechanism unless mineral baths used	Yosipovitch et al. (53); Herrero-Fernandez et al. (58)
Middle Eastern *hammam*	Warm/steam rooms; mechanical exfoliation (gloves); variable mineral contact	Moderate-to-high heat; scrubbing common	May improve cleansing and stress; risk of barrier micro-trauma with coarse exfoliation; potential worsening of AD if vigorous	Heat/steam →↑TEWL; abrasion disrupts SC lipids and microbiome	Herrero-Fernandez et al. (58); Eichenfield et al. (56)
European thermal balneotherapy (e.g., France, Italy, etc.)	Selenium, bicarbonate, silicate waters; regulated protocols in spa settings	Generally moderate temp, 10–15 min sessions; post-bath emollients standard	Reported anti-inflammatory and barrier-restoring effects when protocols are followed; symptom relief of itch/pruritus	Minerals may modulate antigen presentation (e.g., Mg^2+^ effects on Langerhans cells), reduce inflammation, and support the acid mantle; structured exposure prevents overheating	Matz et al. (51); Schempp et al. (52); Weisshaar et al. (57); Eichenfield et al. (56)
Dead Sea salt (Mg-rich) balneotherapy	High magnesium content; often diluted baths at home or on site	Lukewarm to warm; brief soaks	Improved hydration, reduced inflammation, better barrier function reported	Mg^2+^ may stabilize the barrier, reduce inflammation, and influence APCs; salt alters osmotic and microbial conditions	Proksch et al. (50); Schempp et al. (52); Herrero-Fernandez et al. (58)

↑ Increased. AD, atopic dermatitis; APCs, antigen-presenting cells; SC, stratum corneum; TEWL, transepidermal water loss.

In Japan, the practice of bathing in an *onsen* is not only a cultural tradition but also a therapeutic tool widely used by the population. Many individuals travel specifically to visit hot springs and even recreate these baths at home using *onsen*-derived bath additives. On the one hand, some *onsen* waters with acidic pH levels (as low as pH 2.0) have been thought to be irritating for specific skin types; however, some people with AD report symptomatic improvement in their condition after bathing in sulfur and ion-rich waters in certain *onsen* settings, possibly owing to partial restoration of a more acidic skin surface together with bactericidal activity against *S. aureus* (54). On the other hand, as spa waters vary, those containing bicarbonates with a mildly alkaline pH have also been considered soothing and beneficial. Bathing in these waters, often at temperatures exceeding 42 °C, is reported to leave the skin feeling smooth, soothed, and soft for extended periods. All types of baths described should be considered complementary practices to skincare products, accompanied by immediate, appropriate moisturizing routines (55).

In contrast, European thermal waters, mainly those rich in selenium, bicarbonate, and silicates, are traditionally used at lower temperatures and have demonstrated anti-inflammatory and skin barrier-restoring effects, particularly beneficial for AD, but only when applied through regulated protocols, emphasizing moderate water temperatures, limited exposure times (10–15 min), and the application of emollients post-bathing to protect and restore the skin barrier (56, 57) (Table 5).

Nonetheless, it is essential to acknowledge that prolonged exposure to excessive heat can have adverse effects on the skin. High water temperatures may increase TEWL, erythema, and potentially impair skin barrier function (58).

#### 5.2.3 The use of exfoliating tools and stiff fibers

The use of abrasive sponges, exfoliating tools, and stiff fibers for bathing is a common practice in Mexico and other Latin American countries. Historically, Mesoamerican cultures, such as the Aztecs, employed natural fibers and abrasive materials for personal hygiene, a tradition that continues in some communities today. For example, pumice stones are commonly used for exfoliating rough areas, such as the feet, elbows, and hands. However, the daily use of stiff fibers can negatively impact the skin barrier and disrupt the skin microbiome, potentially exacerbating conditions like AD. Alarmingly, this practice often extends to infants, as many mothers still use these fibers to cleanse their babies’ delicate skin, which may lead to increased skin irritation and barrier dysfunction from an early age (59, 60).

Beyond their prevalence in Latin America, similar exfoliation habits are also prevalent across Arab countries. In Morocco and Algeria, the *lifa*—a textured glove—is traditionally used in *hammams* for deep exfoliation. In Libya, a rough sponge or cloth known as a *harsha* is widely employed, while in Egypt, natural fiber exfoliating tools remain an integral part of regular cleansing rituals.

Because published evidence directly linking these practices to AD onset is scarce, several statements in this section are informed primarily by the authors’ regional clinical experience. However, frequent abrasive cleansing may represent a biologically plausible aggravating factor in atopic skin: repeated microtrauma may increase TEWL, facilitate irritant and allergen penetration, and disrupt the skin microbiome, thereby promoting inflammation and perpetuating the itch–scratch cycle. For this reason, patients with atopic skin, particularly infants and children, should be advised to avoid routine abrasive exfoliation and follow gentle cleansing practices.

Understanding cultural hygiene habits is crucial for developing practical skincare recommendations in populations where such practices are prevalent.

### 5.3 High and low latitude regions

Regional factors, particularly latitude, temperature, and humidity, have a significant impact on the prevalence and severity of skin atopy. Several studies have reported higher AD and eczema rates in high-latitude regions with cold, dry climates, such as Northern Europe, Canada, and Japan, where reduced ambient humidity and UV exposure may contribute to skin barrier dysfunction, increased TEWL, and lower vitamin D synthesis (61–63). Low vitamin D levels, common in high-latitude regions, may exacerbate AD severity by modulating Th2 driven inflammation and supporting antimicrobial peptides production (64). In contrast, tropical and low-latitude regions with high humidity often exhibit more variable outcomes. While elevated moisture levels may enhance skin hydration, excessive sweating and microbial overgrowth (e.g., *S. aureus*) can exacerbate atopic symptoms in some individuals (62, 65).

When assessing the role of ambient humidity in atopic skin, it is also important to consider concurrent environmental changes. In some urban settings, air pollutants may interact with atmospheric moisture and contribute to skin irritation or barrier stress, although their specific contribution to AD expression is likely to be context-dependent. The use of air conditioning (AC) systems has steadily increased over the past decades, driven by urbanization and rising temperatures. It reduces indoor humidity and alters air quality, which may adversely affect skin health, particularly in individuals with atopic skin, despite its benefits in providing thermal comfort and heat protection (66–68). In tropical and subtropical countries where AC usage has increased dramatically due to urbanization and rising temperatures, such as in Southeast Asia, the Middle East, and parts of Latin America, the dermatological impact of these systems may be more pronounced. This dryness may be particularly problematic in individuals with already compromised epidermal integrity (69). Moreover, AC systems often recirculate indoor air, potentially accumulating allergens such as dust mites, mold spores, pet dander, and VOCs, all of which have been implicated in triggering or aggravating AD symptoms (65, 70, 71).

### 5.4 Arab and Asian countries: the culture of avoiding the sun

Cultural practices that limit sun exposure can affect individuals’ skin health. The use of full-body clothing in many Arab countries and parasols in several East Asian societies are beneficial habits that protect against photodamage and overheating. However, in some contexts, chronically limited sun exposure may contribute to lower vitamin D status, which could have implications for skin barriers and immune function.

UV radiation, primarily from sunlight, exerts both beneficial and harmful effects on atopic skin, depending on dose, duration, and individual skin reactivity. Controlled UV exposure, particularly narrowband UVB, has demonstrated therapeutic efficacy in moderate to severe AD, improving symptoms by reducing inflammation, modulating immune responses, and enhancing skin barrier repair (72, 73).

Furthermore, moderate natural sun exposure can increase the cutaneous synthesis of vitamin D, which plays a role in skin barrier integrity, immune modulation, and antimicrobial defense (14). The use of traditional Muslim clothing or parasols in some Asian settings may contribute to reduced vitamin D levels in some individuals. Suboptimal vitamin D levels have been associated with increased AD severity in some populations (39, 61, 74, 75).

Vitamin D deficiency affects populations across all geographic regions and socioeconomic levels. It is estimated that over one billion individuals worldwide have insufficient serum levels of 25-hydroxyvitamin D [25(OH)D] (76). Apart from Arab and Asian cultures, the prevalence is particularly high in populations residing at higher latitudes with limited sun exposure. Besides its role in vitamin D synthesis, it enhances antimicrobial peptides production, such as LL37 and βdefensins, which are typically reduced in atopic skin and are crucial for controlling *Staphylococcus aureus* colonization and maintaining microbial balance (77). Thus, vitamin D deficiency remains one of the most common nutritional deficiencies globally (76).

### 5.5 The role of clothing and textile fibers

The interaction between skin and clothing fibers can significantly influence the skin’s condition, particularly in individuals with compromised skin barriers.

Synthetic fibers, even when they are soft and thin, can present challenges for atopic skin across various countries and populations. The physical characteristics of the fibers, such as size and texture, also contribute to their potential irritant effect. Larger fibers with layered structures and a palm-like appearance tend to be more irritating to sensitive skin (78, 79).

Moreover, synthetic fibers have been shown to disrupt the skin microbiota balance, which is a critical factor in the pathophysiology of AD. A particular concern arises with exposure to isocyanates; chemical components found in elastane fibers. These compounds have been shown to interfere with the therapeutic pathways of commensal bacteria and have been associated with an increased prevalence of AD in population-level analyses (80). Specifically, diisocyanates have been found to disturb the microbiota by promoting the growth of *S. aureus*, a bacterium associated with AD exacerbations, while reducing levels of *Roseomonas mucosa* (80). This latter bacterium has been shown to improve the severity of AD by enhancing the skin barrier, inhibiting *S. aureus* growth, and potentially increasing the production of beneficial lipids that support skin health.

Recent evidence indicates that diisocyanates can also activate the transient receptor potential ankyrin 1 (TRPA1) channel in murine models, a mechanism that may contribute directly to the pathophysiology of AD (81).

Despite the documented adverse effects of synthetic materials on the skin, their usage is increasing globally in all regions, often replacing cotton and silk, which are traditionally recommended. Silk, which has shown a positive effect on the skin (82), remains underutilized, likely due to its higher cost and limited accessibility, except in regions where it is produced.

Therefore, the choice of textile fibers is of paramount importance in the management of atopic skin conditions. Not only can fibers directly irritate the skin, but their influence on the microbiota may also exacerbate AD symptoms, highlighting the need for careful consideration of clothing materials in affected patients.

## 6 Social media: an additional element influencing skin atopy

Social media has become one of the most influential tools for disseminating information, and represents a global phenomenon between education and misinformation. Social media presents a significant opportunity for the healthcare and scientific communities, as it provides a lasting communication channel that can effectively deliver educational messages to patients, provided it is used appropriately and relies on evidence-based sources, such as those from healthcare professionals (83). Moreover, this channel may represent a particularly valuable advantage for individuals with limited access to the healthcare system, as it facilitates access to reliable information and guidance on disease management.

Patients and their families often turn to these platforms to seek guidance on the best approaches and products for skin care. However, it is essential to recognize that medical information may at times be inaccurate, incomplete, or biased due to a range of contributing factors. These may include reliance on non-validated sources, misinterpretation or miscommunication of scientific data, selective reporting driven by financial or commercial interests, the use of flawed or partial datasets, and both intentional and unintentional dissemination of misinformation. These elements can substantially compromise the accuracy, validity, and reliability of the medical content shared with the public, ultimately influencing health behaviors, decision-making, adherence, and product or treatment choices that could worsen AD outcomes (84, 85). Such misleading or commercially biased content often leads patients to adopt inappropriate skincare routines or delay evidence based care, thereby aggravating AD symptoms. In addition, interpretations regarding regional patterns of social media use and their dermatologic impact are partly based on expert clinical experience, as comparative data across regions remain limited.

As an example of regional regulatory responses, the United Arab Emirates has introduced a mandatory “advertiser permit” for individuals conducting advertising activities on social media. Breaches of the media law and implementing regulations are subject to attract administrative penalties, including fines that can reach 1 million Arab Emirates Dirhams (86).

Therefore, the impact of social media is global and transcends regional boundaries, making this a worldwide concern that may influence public perception, health behaviors, and the dissemination of medical information on an unprecedented scale.

## 7 Discussion: current and future directions

Despite regional differences in culture, climate, and healthcare systems, the management of atopic skin appears to share several broad principles across countries, particularly regarding barrier-supportive dermocosmetic care and the use of comparable therapeutic approaches. Across both the published literature and the expert discussion, there is broad consensus on the importance of transparent, straightforward product formulations, as well as on the need for patient education and appropriate oversight of dermocosmetic products. For effective management of atopic skin and prevention of future flare-ups, it is essential to use products specifically designed to support and restore the four key skin barrier functions: physical, chemical, immunological, and microbial. Each of these plays a critical role in maintaining skin homeostasis and protecting against environmental and endogenous triggers. A multifunctional approach with the addition of ceramides, fatty acids, humectants and NMFs, inflammatory or immune modulators, and specific prebiotics and postbiotics, not only helps relieve current symptoms, but also strengthens the skin’s resilience and minimizes the likelihood of future exacerbations.

Vitamin D supplementation has shown potential benefits in some studies, including reductions in disease severity and improvements in skin barrier function (77). There are also some promising results on the topical use of vitamin D analogs and Pro-vitamin D ingredients, which enhance the natural production of vitamin D (87, 88). These may also be considered as they may exert beneficial effects on skin barrier function, including modulation of keratinocyte differentiation, antioxidant protection, enhanced lipid synthesis, improved hydration, and support for epidermal integrity (89). Thus, ensuring adequate vitamin D levels can be a valuable adjunctive strategy in the comprehensive management of atopic skin.

Environmental and lifestyle factors may play a crucial role in the optimal management of the disease, although their relative impact varies across regions and is informed by both published evidence and clinical experience. Practical recommendations generally include limiting bathing frequency and water temperature, avoiding abrasive materials, and recognizing the potentially harmful effects of over-cleansing on the skin barrier and microbiome. On the other hand, some bath practices can offer an interesting complementary approach to skincare products, allowing the incorporation of bath additives. Practical counseling points across the major exposures discussed are summarized in Figure 1.

In air-conditioned environments, maintaining skin hydration is essential and can be optimized through regular moisturizer use, indoor humidification, and allergen control via filter maintenance.

Regarding the use of textile fibers, cotton and silk are generally considered safe and are recommended. Coarse wool, despite being a natural fiber, is known to cause itching and is therefore not recommended. Modern textile manufacturing techniques offer advantages for individuals with atopic skin, particularly through the development of finer and smoother fibers, such as superfine and ultrafine merino wool, which are well tolerated and may help reduce irritation (90). Other technologies, such as silver-coated fabrics, chitosan, or cellulose, have shown promise in reducing AD severity and *S. aureus* colonization (79).

An important consideration is that all the factors discussed—genetic, environmental, and cultural—interact within a multifactorial, complex framework, often influencing one another. Consequently, the impact of a single factor may vary depending on the context created by other interacting variables. For instance, the effect of a specific humidity level may differ depending on different environmental parameters, such as UV radiation or temperature. This interplay highlights the importance of evaluating these factors not in isolation, but rather in terms of their combined, context-dependent effect on AD. Within this multifactorial context, vitamin D emerges as a crosscutting factor whose effects extend beyond metabolism to include immunomodulation relevant to atopic skin. Low vitamin D levels, more common in high latitude regions and in populations with culturally limited sun exposure, may therefore amplify both barrier and immunological vulnerabilities. Integrating this dimension helps explain part of the regional variability observed in atopic skin expression.

When treating atopic skin, it is essential to recognize that patients with intrinsic risk factors—such as genetic background, ethnicity, age, or systemic conditions—may be more vulnerable to barrier dysfunction and less able to adapt to external stressors. These non-modifiable variables weaken the epidermal barrier and limit the ability of the stratum corneum to adapt to external stressors. If the combined impact of intrinsic and extrinsic stressors surpasses the skin’s repair capacity, natural restoration may fail. This increased vulnerability heightens the risk of ongoing barrier dysfunction and symptom flare-ups, making it essential to consider this complexity in the clinical management of AD (4).

Social media has become one of the most influential channels for information sharing and can be a valuable tool for patient education about atopic skin conditions. However, healthcare professionals remain essential for providing accurate, evidence-based guidance. Increasingly though, their efforts are not enough to offset the overwhelming amount of often misleading or incorrect content on social media. This trend may pose a serious threat to public health, as misinformation can shape health behaviors and potentially worsen disease outcomes. Its extensive reach requires coordinated international responses and action from public health authorities and regulators to curb the spread of false information. In addition, health misinformation adds strain to healthcare systems by increasing unnecessary visits, treatments, and mistrust. Addressing this challenge is important to protect public health and maintain healthcare infrastructure.

We are at a historic turning point in the treatment of AD, with biological therapies and small molecules leaving conventional treatments behind in many cases worldwide. Nevertheless, it remains important to remember that patients with atopic skin also require basic supportive measures, including appropriate hygiene, regular moisturization, and individualized counseling on the exposures outlined herein, alongside access to emerging therapies and essential daily skin care practices. Figure 1 also highlights practical approaches to mitigate these exposures and support barrier repair, emphasizing actionable strategies for patients and clinicians.

This narrative review is subject to several limitations. First, by design, it relied on a pragmatic rather than systematic literature search and is therefore vulnerable to non-systematic selection bias. Second, the author focus group was purposively assembled to reflect experience from diverse climates and cultural settings, but it was not intended to be globally representative; accordingly, some regions, practices and perspectives may be underrepresented. Third, the expert-input process was used to support interpretive synthesis rather than formal consensus building. No Delphi methodology, quantitative voting, or prespecified consensus thresholds were applied, and portions of the regional interpretation presented in Sections “3 Why environmental and regional factors may have an impact on atopic skin: the four skin barriers.” “4 Regional intrinsic factors impacting atopic skin,” “4.1 Genetic component: filaggrin mutations across regions,” “4.2 Consanguinity: cultures that promote intrafamilial marriage” and “5 Regional extrinsic factors impacting atopic skin” therefore reflect expert clinical experience where robust comparative evidence is limited. Minor points arising during manuscript development were resolved by discussion and subsequent e-mail exchange among the authors. Finally, medical writing support was sponsor funded; however, all decisions regarding content, interpretation, and submission were made by the authors, and the sponsor had no role in literature selection, analysis, or conclusions. Despite these constraints, we believe this synthesis provides an honest, pragmatic, and regionally grounded perspective that may help inform more context-appropriate care for patients with atopic skin.

To conclude, atopic skin represents a complex and multifactorial condition, influenced by the interplay between genetic-ethnic, immune, environmental, cultural, and psychosocial factors. This complexity underscores the need for a holistic, individualized approach to management, one that targets not only clinical symptoms but also underlying triggers and the patient’s lifestyle. Therefore, integrating dermatological, environmental, and behavioral perspectives is essential to improving long-term outcomes and supporting patients more effectively. In this context, the incorporation of structured patient and caregiver education programs within healthcare systems, which the authors have already implemented, emerges as a valuable tool to empower individuals, enhance treatment adherence, and foster better disease control.
