# Supplementary material for: Understanding worldwide skin atopy across regions. Environmental, cultural, genetic, and lifestyle factors
Source: Front Med (Lausanne). 2026 Apr 1;13:1796337. doi: 10.3389/fmed.2026.1796337 (PMC13079169; doi:10.3389/fmed.2026.1796337)
Supplement: Supplementary file 1 [file Table_1.docx]

# Supplementary Table S1. Pre-meeting guiding questions used to structure the focus-group discussion

The questions below were circulated to the authors before the online meeting to guide preparation and structure the thematic discussion.

| **Discussion Domain** | **Guiding Questions** |
| --- | --- |
| **Patient and disease characteristics** | - Prevalence of AD in the region, % of patients with atopic skin - Characteristic clinical patterns of atopic skin - Typical age ranges - Skin phototype (Fitzpatrick I–VI) - Common skin features (e.g., sensitive, dry) - Other related factors (e.g. allergies, genetics) |
| **Environmental and lifestyle factors** | - Impact of local climate (weather, humidity) on atopic skin - Environmental factors (pollution, water quality, pollen/allergens, fabrics/clothing) - Lifestyle factors (sleep, smoking, diet) - Psychological/hormonal factors  - Other relevant environmental influences |
| **Cultural habits and skin care practices** | - Bathing and hygiene practices (frequency, shower/bath, soaps/oils, cleansing tools, temperature)  - Typical skincare routine (product types and frequency of use)  - Patient preferences and cultural expectations regarding skincare products |
| **Management approaches** | - Commonly prescribed/recommended treatments  - First- and second-intention approaches (pharmacological treatments and dermocosmetic measures)  - Traditional practices or remedies relevant to skin care  - Treatment goals during flares and remission  - Quality-of-life impact and psychosocial effects  - Criteria for choosing dermocosmetics |
| **Unmet needs and practical considerations** | - Unmet needs (education, products, etc.)  - Ideal dermocosmetic characteristics  - Additional recommendations relevant to product development |

AD: atopic dermatitis
